# On Four Types of Devaluation of Outcomes Due to Their Costs: Delay, Probability, Effort, and Social Discounting

**DOI:** 10.1007/s40732-019-00340-x

**Published:** 2019-03-22

**Authors:** Wojciech Białaszek, Paweł Ostaszewski, Leonard Green, Joel Myerson

**Affiliations:** 1 SWPS University of Social Sciences and Humanities, Chodakowska 19/31, 03-815 Warszawa, Poland; 2 Washington University, St. Louis, MO, USA

**Keywords:** Delay discounting, Probability discounting, Effort discounting, Social discounting, Magnitude effect, Factor analysis

## Abstract

Discounting refers to decreases in the subjective value of an outcome with increases in some attribute of that outcome. The attributes most commonly studied are delay and probability, with far less research on effort and social discounting. Although these attributes all represent costs that reduce subjective value, it is as yet unclear how the extent to which they do so is related at the individual level. Accordingly, the present study examined the degree to which individual participants discounted hypothetical monetary rewards on each of four discounting tasks in which the delay, probability, effort, and number of people with whom the money was to be shared were manipulated. At the group level, larger amounts were discounted less steeply than smaller amounts when delay and effort were varied, whereas larger amounts were discounted more steeply when probability and number of people were varied. At the individual level, the correlational pattern was examined using exploratory factor analysis. A six-factor structure (with separate factors for delay and effort, and two factors each for social and probability discounting) described the relations among indifference points. At a more molar level, a two-factor structure, which corresponded to the direction of the observed magnitude effects, described the relations among area-under-the-curve measures of discounting in the eight conditions resulting from crossing two monetary amounts with the four cost factors. We conclude that despite sharing some similarities, individual and group differences in discounting involving the different types of costs reflect mostly separate processes and traits.

Consider a situation in which you could receive something you want after a delay but its delivery is not certain. Furthermore, you are required to engage in some effort to obtain the outcome, and once it is received, you have to share it with others. Such a situation occurs when we have to wait to receive money; its receipt is not always certain, sometimes we have to exert some effort to receive it, and we often have to share it with other people. Each of these four cost factors, delay, risk, effort, and a social component of sharing with others, decreases the subjective value of the outcome.

Such decreases in subjective value have been studied in the context of discounting (Rachlin, 2006). Rachlin (1993; Rachlin & Raineri, 1992) noted that outcomes lose value (i.e., their value is discounted) when they are delayed, their receipt is not certain, or if they are to be shared with others. Later studies added a fourth cost factor to discounting, namely the effort needed to obtain a reward (Białaszek, Marcowski, & Ostaszewski, 2017; Mitchell, 1999, 2003; Ostaszewski, Bąbel, & Swebodziński, 2013; Sugiwaka & Okouchi, 2004). A question of both theoretical and practical importance is whether these cost factors engage the same or different underlying processes and traits (Myerson, Green, Hanson, Holt, & Estle, 2003). To answer this question, the present study takes two approaches. The first approach examines the effects of experimental manipulations based on the assumption that if a specific manipulation affects two dependent variables in the same way, then the same processes may be involved in both cases, but that if that manipulation affects the two dependent variables differently, then different processes may be involved. The second approach examines individual differences and the intercorrelations among the different discounting types, using factor analysis to determine the number of discounting factors.

Discounting can be measured using indifference points to estimate the subjective value of a reward. That is, one can estimate the amount of reward that a person judges approximately equal in value to that of another (usually larger) reward amount, the acquisition of which depends on some cost factor. In the case of delay discounting, for example, one may determine the smallest amount of immediate reward exchangeable for a larger, delayed reward. If the delayed outcome is, say, PLN 150 (i.e., 150 new Polish złotys) in one month and the immediate one is also PLN 150, the typical choice will be the immediate reward. If the immediate amount is systematically decreased, however, at some point the participant may prefer the delayed alternative. It is important to note that this point may be different for every participant: some may switch their choice to the delayed alternative when the immediate amount is only slightly smaller (e.g., PLN 145), indicating shallow discounting, whereas others may continue to choose the immediate reward until it is decreased below PLN 50, indicating they discounted the larger amount steeply because it was delayed and they chose not to wait, even for the additional PLN 100.

Steep discounting of delayed rewards is associated with an array of maladaptive behaviors, and with addiction in particular. A meta-analysis by MacKillop et al. (2011; see also Amlung, Vedelago, Acker, Balodis, & MacKillop, 2017) found that individuals with an addiction discounted delayed rewards more steeply than control groups. Discounting of probabilistic rewards, however, is less predictive. Some studies report that substance use and abuse is related to greater probability discounting (Reynolds, Richards, Horn, & Karraker, 2004; Yi, Carter, & Landes, 2012), whereas others have failed to find such a relationship (Reynolds, Karraker, Horn, & Richards, 2003; Takahashi, Ohmura, Oono, & Radford, 2009). Very few studies have examined the relations between addiction and effort or social discounting, although a study of methamphetamine users found that they showed steeper social discounting, which involves sharing rewards, than controls (Yi et al., 2012). Further research is needed, and such research should include not just studies examining the different types of discounting separately, but also studies that examine more than one type of discounting in order to determine the relations among the effects of different cost factors. We believe that assessing the extent to which an individual’s tendency to discount delayed rewards predicts their tendency to discount effortful rewards, for example, is essential if we want to explain the traits and processes underlying problematic behaviors.

## Amount-Dependent Discounting

The best-known example of a magnitude effect is the finding that larger delayed reward amounts are discounted less steeply than smaller ones (e.g., Green, Myerson, Oliveira, & Chang, 2013; Kirby, 1997; Thaler, 1981). In this example, the cost factor, delay, reduces the relative subjective value of a larger reward less than it reduces the relative subjective value of a smaller reward. The magnitude effect has been observed not only with respect to delayed monetary payoffs but also with other delayed outcomes including health (Chapman, 1996) and consumable rewards as diverse as candy and vacations (Estle, Green, Myerson & Holt, 2007; Raineri & Rachlin, 1993). Much less is known about the magnitude effect in effort discounting, but it appears to be similar to that in delay discounting. For example, Ostaszewski et al. (2013; see also Białaszek et al., 2017) found that larger reward amounts were discounted less steeply than smaller amounts on tasks in which the amount of physical effort was varied as well as on tasks that varied in the amount of cognitive effort required.

A reverse magnitude effect, in which larger amounts of reward are discounted more steeply than small amounts, is consistently observed in probability discounting (Green, Myerson, & Ostaszewski, 1999; Myerson, Green, & Morris, 2011). A reverse magnitude effect also is observed with social discounting: larger rewards to be shared with others lose a greater proportion of their value as the number of people with whom the reward is to be shared increases (Ostaszewski & Osiński, 2011) or as their social distance increases (e.g., from friends to strangers; Rachlin & Jones, 2008a). Thus, the effects of the reward amount appear to divide these types of discounting into two categories, depending on whether larger amounts are discounted more or less steeply than small amounts.

## Correlation Analyses and Factor Analytic Approach

The majority of studies investigating the relations among different types of discounting have focused on delayed and probabilistic rewards, perhaps initially because time and risk are dimensions that were strongly linked theoretically. If one dimension or one trait (e.g., impulsivity) underlies the other, then delay and probability discounting should be strongly correlated. On the contrary, researchers have typically found either no or weak correlations between rates of delay and probability discounting (Holt, Green, & Myerson, 2003; Mitchell, 1999; Myerson et al., 2003; Ohmura, Takahashi, Kitamura, & Wehr, 2006; Shead & Hodgins, 2009). The literature on the relation between other types of discounting is relatively sparse, although a bit more is known about social discounting than about effort discounting (cf. Mitchell, 1999). Rachlin (2006) noted parallels between delay and social discounting, and indeed, some studies report a positive correlation between them (Rachlin & Jones, 2008a) as well as correlations among delay, social, and probability discounting (Jones & Rachlin, 2009), although these were rather modest.

It is perhaps surprising that only a few studies have examined the factor structure of different discounting tasks, and those concerned delay and/or probability discounting. Green and Myerson (2013) showed that delay discounting and probability discounting of monetary payoffs and consumable goods loaded on two separate factors linked not to the nature of the rewards but to the type of discounting. In contrast, other research suggests that individual differences in probability discounting (Terrell, Derenne, & Weatherly, 2014) and delay discounting (Weatherly, Terrell, & Derenne, 2010) may be outcome-specific. Although factor analysis has been relatively neglected to date in studies of discounting, it is commonly used to study individual differences in personality and seems particularly well suited to address the issue of how many different traits are involved in the various types of discounting.

## Aims and Scope of the Present Study

There are multiple parallels among at least some different types of discounting. There are consistencies between magnitude effects both between delay and effort discounting (where discounting decreases with amount), and between probability and social discounting (where the opposite is true). The lack of direct comparisons among the same participants engaged in all four types of discounting, however, led us to conduct the present study, the main purpose of which was threefold: (1) to determine how amount of reward affects delay, probability, effort, and social discounting; (2) to investigate correlations among the four types of discounting; and (3) to explore the underlying factor structure of discounting and its implications for individual differences in choice and decision making.

## Method

### Participants

We recruited 160 participants from the local community in accordance with university ethics committee regulations. Participants (82 males and 78 females) ranged in age from 21 to 60 years (M = 35.74; SD = 10.45).

### Procedure

After signing an informed consent form, participants provided basic demographic information and then proceeded to the discounting tasks. The discounting tasks used a fixed-choice procedure (Rachlin, Raineri, & Cross, 1991) to estimate indifference points, where an indifference point is the amount of money available without any cost that is subjectively equal in value to the undiscounted amount accompanied by a specified cost.

Participants were provided a 24-page response booklet that was used to determine 24 indifference points (4 discounting types, 2 reward amounts, and 3 cost values), one per page. A heading at the top of each page specified the nature of the choice alternatives (e.g., “gain immediately or in 6 months”). Below the heading were two columns. In the left column, amounts corresponding to potential indifference points were presented in descending order, beginning with the actual amount of the to-be-discounted reward and ending with PLN 0 in 30 decrements. Sample headings for the three other discounting tasks are “gain for sure or with a 45% chance, “gain to be kept for yourself or to be shared with five strangers,” and “gain without an effort or after climbing to the 11^th^ floor.” Note that, following Rachlin (1993) and Rachlin and Raineri (1992), the social discounting cost was defined as the number of people with whom a reward was to be shared.

All reward amounts and cost values across the 24 experimental conditions are presented in Table 1. The four types of discounting tasks were presented in a counterbalanced order, and within each task, the two amount conditions (small or large) of the to-be-discounted reward were counterbalanced. For each amount in each discounting task, three indifference points were estimated, one for each cost value. The cost values always were presented in an ascending order (e.g., 1 month, then 6 months, and then 2 years). Different reward amounts were used in each discounting task to make the experimental session less monotonous for participants and to reduce the likelihood of repetitive responses.

### Data Analysis

To answer the question of whether the four discounting types reflect the same underlying mechanisms and traits or involve separate mechanisms and traits, we used a three-step approach. First, we examined whether the different types of discounting are similarly affected by the amount of reward. Second, we analyzed the intercorrelations among the four discounting types. Third, we performed exploratory factor analyses in order to determine whether the associations among the various discounting conditions can be described by a set of more fundamental variables (factors). Two factor analyses were performed, one on the indifference points (expressed as proportions of the to-be-discounted amount) and the other on the areas under the curve (*AuC*s; Myerson, Green, & Warusawitharana, 2001). Indifference points were estimated as the last amount of immediate, certain, effortless, or unshared reward that a participant chose in each condition.

## Results

### Amount-Dependent Discounting

**Fig. 1:**
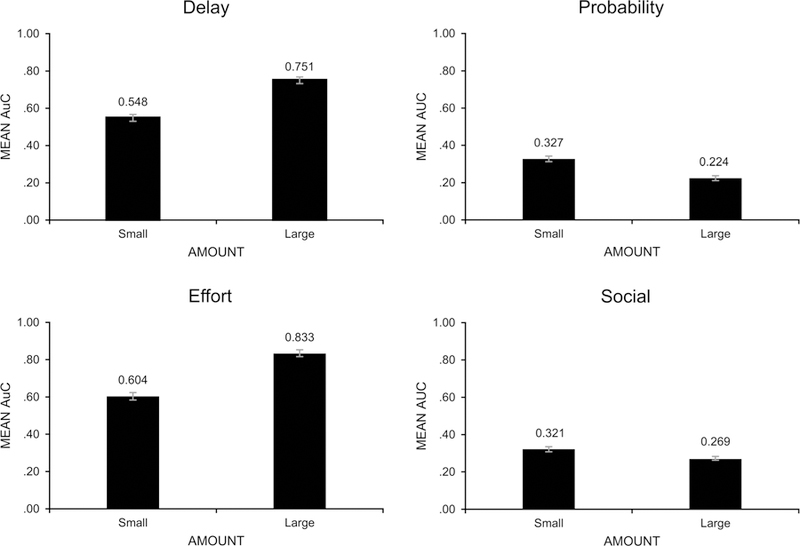
Mean Areas under the Curve (AuC) for small and large reward amounts for each of the four types of discounting. A magnitude effect is present for delay and effort discounting, and a reverse magnitude effect is present for probability and social discounting. Error bars represent ±1standard error of the mean

To determine whether amount of reward has the same effect on different types of discounting, we performed *t*-tests for dependent samples on the AuC values for the small and large reward conditions (shown in Figure 1). These tests revealed significant magnitude effects for delay (*t*(159) = 12.11; *p* < .001; *d* = 0.96) and effort discounting (*t*(159) = 12.94; *p* < .001; *d* = 1.02) in which smaller rewards were discounted more steeply, and significant reverse magnitude effects for probability (*t*(159) = 7.64; *p* < .001; *d* = 0.60) and social discounting (*t*(159) = 4.56; *p* < .001; *d* = 0.36) in which smaller rewards were discounted less steeply. Following Cohen (1988), these effect sizes can be interpreted as large for delay and effort discounting, medium for probability discounting, and small for social discounting.

### Correlations among Discounting Types

Moderate positive correlations (.54 to .59; all *p*s < .001) were observed between the AuCs for different amount conditions within the same discounting type (e.g., probability PLN 150 and PLN 30,000: *r* = .55). All other correlations between different discounting conditions were lower, implying greater similarity within types of discounting than between types.

As may be seen in Table 2, half of the correlations among the AuCs for different discounting conditions were significant, and half were not. It should be noted that delay and effort discounting were significantly correlated at all four combinations of reward amounts (small–small, large–large, and the two small–large combinations), and for probability and social discounting, three of the correlations among amount conditions were significant, suggesting that two separate factors may underlie individual differences in discounting at this level of analysis. In contrast, delay and probability discounting conditions were never significantly correlated. The other combinations of conditions from different types of discounting (i.e., delay and social, effort and social, effort and probability) produced intermediate results (one or two significant correlations).

### Factor Analyses

Factor analyses were conducted at two levels, one based on the eight AuCs just discussed, and the other based on the 24 indifference points corresponding to the different cost factor conditions. To determine the number of factors in exploratory factor analysis of the indifference points, we relied on Velicer’s (1976) minimum average partial method (MAP), which is based on mean partial correlations between variables (O’Connor, 2000). This method identified a six-factor solution as best; the smallest mean square of partial correlation was achieved in the sixth step and equaled 3.472*10^−2^. Similar conclusions follow from Cattell’s (1966) scree plot analysis, which suggested a six- to eight-factor solution, and Kaiser’s (1974) criterion (eigenvalue greater than 1.0), which resulted in a seven-factor solution.

The factor analysis of indifference points was conducted using the principal axis extraction method with Oblimin rotation and an initial value of delta = 0, not imposing any artificial ad-hoc correlations between factors, as recommended by Fabrigar, Wegener, MacCallum, and Strahan (1999). Oblique rotation takes into account that the factors do not have to be orthogonal and, in theory, could be correlated. The Kaiser-Meyer-Olkin (KMO) measure of sampling adequacy was .692, exceeding the threshold of .5, and Bartlett’s test of sphericity was significant (*Χ*^*2*^(276) = 2148.70; *p* < .001).

Overall, the six factors seen in Table 3 explained 58.33% of the variance. All factor loadings above .32 are bolded in the pattern matrix, following Tabachnick and Fidell’s (2007) recommendation that only loadings higher than that should be interpreted. The first factor explained 18.56% of the variance, and the following five factors explained 16.97%, 7.91%, 6.34%, 4.71%, and 3.83%, respectively. The first factor is composed of all six conditions of the delay discounting task (i.e., all three delays crossed with the small and large amounts). The second and sixth factors correspond to social discounting of small and large amounts, respectively. The third and fifth factors represent different probability discounting conditions, with small and large amount conditions loading on each factor, and the fourth factor is composed of all six conditions of the effort discounting task. These analyses show high consistency in the loading of different types of discounting on separate factors as well as clear discriminability between factors. It is interesting that delay and effort discounting each formed a factor that included both reward amounts, whereas different amount conditions of social discounting and probability discounting loaded on separate factors (see Table 3).

In addition, an exploratory factor analysis was performed on the AuC measures in order to explore the structure of discounting factors at this higher level. This analysis, however, may have limitations. It is important to note that the MAP test (Velicer, 1976) did not provide a coherent solution (i.e., it could not reliably identify a clear factor structure for the AuC measures), which might be because there was more unsystematic than systematic variance (O’Connor, 2000). Therefore, we based our decision on how many factors to retain on the simplest criterion, the change in the eigenvalue with each additional factor, as represented in Cattel’s scree plot. After the first two eigenvalues (2.201 and 2.104), the third was 1.187, and the fourth was 0.873. Given the large reduction in eigenvalues and the correlational results described previously, we decided to retain a two-factor solution.

Again, the basic assumptions were met (the KMO equaled 0.595), and Bartlett’s test of sphericity was significant (*Χ*^2^(28) = 323.655; *p* < .001). The average communality after extraction was .390 (with a minimum of .242 and maximum of .610). Despite the low communality for the large reward condition of probability discounting (i.e., .242), we decided to include this data in further analyses because of their exploratory nature and the theoretical importance of decisions involving large, probabilistic rewards.

The two-factor solution for the AuC data explained 39.02% of the variance (20.23% by the first factor) and provided an interpretable solution. The first factor was composed of the AuCs for both amount conditions of the delay discounting task and the AuCs for both amount conditions of the effort discounting task. The second factor was composed of the AuCs for both amount conditions of the probability and social discounting tasks (see Table 4). There were no cross loadings between the two factors. It is interesting that the first factor involves types of discounting that showed strong magnitude effects, and the second factor involves types of discounting that showed reverse magnitude effects, although these were somewhat weaker. The general finding of both factor analyses is that the different types of discounting are not reducible to a single process or trait, consistent with the different effects of reward amount on discounting and the patterns of correlations among the variables.

## Discussion

The goal of the present study was to examine similarities and differences among four different types of discounting (i.e., delay, probability, effort, and social) defined by different cost factors that can each result in devaluation of rewards. It is important to note that the findings indicate that more than one mechanism underlies the discounting of large and small rewards, and more than one trait underlies individual differences on the various types of discounting.

With respect to the underlying mechanism(s), we would note that the amount of reward had different effects depending on the type of task. Consistent with previous findings, small delayed rewards were discounted more steeply than large ones, whereas large probabilistic rewards were discounted more steeply. The effects of amount on effort discounting paralleled those with delay discounting, and the effects of amount on social discounting, although weaker, paralleled those with probability discounting. It is interesting that analysis of the AuCs revealed that tasks that showed similar amount effects loaded on the same factors (i.e., delay and effort on Factor 1, and probability and social on Factor 2; see Table 4). The present finding of a correspondence between type of discounting and factor loading has not been reported previously, but it fits neatly with the previous finding that whereas delay and probability discounting load on separate factors, the discounting of losses, which does not show amount effects, loaded on yet a third factor (Mejía-Cruz, Green, Myerson, Morales-Chainé, & Nieto, 2016). Taken together, these results suggest that amount effects may reflect something more fundamental about the different types of discounting than just their susceptibility to the effect of amount.

Exploratory factor analyses were conducted at both more molecular and more molar levels (i.e., indifference points and AuCs, respectively). Although different factors were observed in these analyses, in both cases the results are clearly inconsistent with a single factor (e.g., impulsivity) underlying all types of discounting. In part, the results of the analysis of indifference points may be thought of as assessing the internal consistency of the four tasks, and the results revealed that two of the discounting tasks, delay and effort, showed higher consistency than the others, with all six indifference points from the delay discounting task loading on one factor, and all six points from the effort discounting task loading on another (i.e., Factors 1 and 4, respectively). The other two tasks showed less consistency, with the indifference points from each task loading on two separate factors (see Table 3). Although one is tempted to interpret the fact that the small amount indifference points from the social discounting task loaded on one factor and the points from the large amount conditions loaded on another, the fact that the indifference points from the probability discounting task loaded on separate factors without regard for reward amount suggests that further research with larger samples and different procedures for estimating indifference points may be warranted before engaging in too much speculation.

As noted, exploratory factor analyses of the AuCs, which are effectively weighted averages of the indifference points from each amount condition of each task, thereby potentially reducing measurement error, revealed a much simpler structure at the molar level, although, again, the structure is inconsistent with a single underlying trait or mechanism. And again, further research with larger samples and different estimation procedures would be desirable, as would the use of confirmatory factor analysis in future studies, particularly because of the implications of the present findings for treatment interventions. Although individual differences in discounting rates are related to various maladaptive behaviors (Madden & Bickel, 2010; MacKillop et al., 2011), the existence of multiple factors implies that an intervention that successfully targets one type of discounting does not guarantee a similar change in other types of discounting. Nevertheless, the existence of two factors at the more molar AuC level of analysis is encouraging with respect to transfer at least between delay and effort discounting on the one hand, and between probability and social discounting on the other.

One concern, however, is that compared to the delay and probability discounting tasks, the social discounting and effort discounting tasks used here may not be as representative, which might affect transfer. For example, one can exert cognitive or physical effort (Białaszek et al., 2017; Ostaszewski et al., 2013), or even both kinds of effort at once. Likewise, in social discounting, when the reward is shared with other people, these others can be relatives, friends, or strangers, and their social distance as well as their number affects the degree of discounting (Rachlin & Jones, 2008b; Osiński, Ostaszewski & Karbowski, 2014). Therefore, research is needed to examine the degree to which the present findings transfer to other instances of these types of discounting. At the same time, other aspects of the present tasks may facilitate transfer. For example, effort discounting often involves delay discounting, as in the scenario studied in the present experiment. Climbing more stairs typically requires more time. Although this is in some sense a confound, it is nonetheless a characteristic of many situations that people find themselves in every day, particularly those involving iterative tasks, and one that might be exploited in efforts to reduce maladaptive behavior.

The present findings suggest that individual differences in different types of discounting reflect largely separate factors or traits, but it also is possible to capture the different processes involved using a single model. For example, Rachlin (1993) proposed a three-component model of discounting in which the subjective value of an outcome is based on three processes: delay, probability, and social discounting. This model may be extended by adding additional discounting processes. For example, incorporating effort, Rachlin’s original model becomes:

(1) $$V=\frac{A}{\left(1+kD\right)\left(1+h\Theta \right)\left(1+sN\right)\left(1+lE\right)}$$

where *V* is the subjective value of a reward of amount *A*, and the letters *D*, *Θ*, *N*, *E* stand for delay, probability (as odds against, or *Θ*), the number of people with whom the reward is to be shared, and the measure of effort, respectively. The corresponding discounting parameters are represented by *k, h, s*, and *l*. (It also is possible, of course, that different exponents may be associated with the different expressions in the denominator, as in Vanderveldt, Green, & Myerson, 2015.)

It should be emphasized that the fact that different types of discounting can be combined into a single equation to describe the interactions among different cost factors does not mean that a single mechanism underlies the different types or that they involve the same or correlated traits. The opposite amount effects found with delayed and probabilistic rewards are evidence that different mechanisms are involved in at least these two types of discounting, and there also is evidence that they involve separate traits. Jones and Rachlin (2009) found that delay, probability, and social discounting were positively correlated, and Mitchell (1999) reported weak but positive correlations between delay, probability, and effort discounting. However, using multiple measures of delay and probability discounting, others have found that they load on separate factors (Green & Myerson, 2013; Mejía-Cruz et al., 2016), and they also loaded on separate factors in the present study, indicating that these two types of discounting involve separate traits. In any case, it should be noted that positive correlations between delay and probability discounting are the opposite of what would be expected if both reflected a single impulsivity trait, because whereas steep delay discounting could be construed as reflecting impulsivity, steep probability discounting corresponds to risk aversion rather than risk taking (Green & Myerson, 2010).

To examine the relations among different types of discounting, the present study used hypothetical monetary rewards as the outcome in each case so as not to confound type of discounting with kind of reward. However, the relations among the discounting of different rewards are also of considerable interest and bear on the question of how many mechanisms and traits underlie the phenomena collectively termed “discounting.” Although some researchers have highlighted what is domain-general in delay discounting, as revealed by positive correlations among discounting of different outcomes (e.g., Odum, 2011), others have pointed out that delay discounting is often domain-specific (Chapman, 1996; Jimura et al., 2011; for a review, see Green & Myerson, 2013), and it has been suggested that probability discounting is also domain-specific (Terrell et al., 2014). We need to know whether someone who is a steep discounter when making decisions involving one type of cost factor and kind of outcome will also be a steep discounter when the decisions involve other cost factors and commodities.

Regardless of how many traits are involved in different types of discounting, we think it is necessary to study combinations of different discounting cost factors, rather than studying each one in isolation. After all, everyday choice situations often involve outcomes that are not only delayed and probabilistic but also require effort to obtain, and once obtained, they may be shared with others. For this reason alone, not to mention the theoretical significance of the interactions and correlations among the types of discounting, and regardless of the number of mechanisms and traits involved, these situations demand attention in future studies.

## Figures and Tables

**Table 1.** Amounts of reward and costs for each discounting task

Discounting task	Small reward	Large reward	Small cost	Medium cost	Large cost
delay	PLN 200	PLN 40,000	1 month	6 months	2 years
probability	PLN 150	PLN 30,000	98%	45%	3%
effort	PLN 100	PLN 20,000	3^rd^ floor	11^th^ floor	40^th^ floor
social	PLN 50	PLN 10,000	2 people	5 people	12 people

**Table 2.** Intercorrelations among AuC measures

	delay PLN 200	delay PLN 40,000	probability PLN 150	probability PLN 30,000	effort PLN 100	effort PLN 20,000	social PLN 50	social PLN 10,000
delay PLN 200	1							
delay PLN 40,000	.59**	1						
probability PLN 150	0.05	0.04	1					
probability PLN 30,000	−0.03	−0.09	.55**	1				
effort PLN 100	.19*	.38**	−0.01	−0.02	1			
effort PLN 20,000	.17*	.35**	−.23**	−.21**	.55**	1		
social PLN 50	.20*	0.08	.31**	0.13	−.16*	−0.04	1	
social PLN 10,000	.23**	0.10	.25**	.24**	−0.12	−0.02	.54**	1

Note: Correlations between the two reward amounts of the same discounting task appear in bold

* *p* < .05

** *p* < .001

**Table 3.** Factor structure of the indifference points obtained from the four discounting tasks. Factors are presented in descending order of the variance accounted for. Within factors, factor loadings also are presented in descending order

Factor	Condition (reward amount and cost)	Factor					
		1	2	3	4	5	6
1. Delay	PLN 200, 6 months	**.815**	−.021	−.046	−.123	−.005	.142
	PLN 40.000, 2 years	**.784**	−.009	.077	.084	.076	−.046
	PLN 200, 2 years	**.739**	−.044	.025	−.074	−.036	.164
	PLN 200, 1 month	**.736**	.042	−.129	−.028	.056	.035
	PLN 40.000, 6 months	**.727**	.023	.008	.207	−.002	−.106
	PLN 40.000, 1 month	**.568**	.034	.057	.179	−.093	−.137
2. Social-A	PLN 50, 5 people	.027	**.887**	.005	.013	−.027	.137
	PLN 50, 12 people	.017	**.817**	.070	.003	.030	.122
	PLN 50, 2 people	−.033	**.680**	.029	−.009	−.016	.090
3. Probability-A	PLN 150, 3%	.029	.114	**.886**	−.033	−.123	.003
	PLN 30.000, 3%	−.084	−.117	**.646**	−.012	.072	.152
	PLN 150, 45%	.012	.252	**.584**	−.048	.231	−.067
4. Effort	PLN 20.00, 40th floor	−.022	−.042	−.137	**.801**	−.052	.185
	PLN 100, 11th floor	.044	−.098	.154	**.760**	−.052	−.034
	PLN 20.000, 11th floor	−.025	.101	−.227	**.708**	.004	.024
	PLN 100, 40th floor	.080	−.221	.218	**.591**	.044	.030
	PLN 100, 3rd floor	−.012	.133	.008	**.545**	.038	−.085
	PLN 20.000, 3rd floor	.177	.002	−.091	**.390**	.034	−.086
5. Probability-B	PLN 30.000, 98%	−.080	−.074	−.160	.056	**.866**	.076
	PLN 30.000, 45%	−.074	−.051	.250	−.036	**.597**	.175
	PLN 150, 98%	.154	.105	.058	−.023	**.564**	−.165
6. Social-B	PLN 10.000, 5 people	.098	.152	.041	−.003	−.011	**.842**
	PLN 10.000, 12 people	.054	.139	.009	.068	−.005	**.764**
	PLN 10.000, 2 people	.063	.145	.077	−.024	.071	**.442**

Note: Factor loadings over .32 appear in bold; letters A and B refer to factors that do not include all six reward and cost conditions of a discounting task

**Table 4.** Factor structure of AuC measures obtained from the four discounting tasks. Factor 1 corresponds to delay and effort discounting, and Factor 2 corresponds to social and probability discounting. Within factors, factor loadings are presented in descending order

	Factor 1	Factor 2
AuC_delay_ PLN 40,000	**.776**	.140
AuC_delay_ PLN 200	**.586**	.278
AuC_effort_ PLN 20,000	**.577**	−.237
AuC_effort_ PLN 100	**.538**	−.172
AuC_social_ PLN 10,000	.117	**.630**
AuC_social_ PLN 50	.087	**.608**
AuC_probability_ PLN 150	−.070	**.591**
AuC_probability_ PLN 30.000	−.148	**.461**

Note: Factor loadings over .32 appear in bold
